# Trade-offs between cost and accuracy in active case finding for tuberculosis: A dynamic modelling analysis

**DOI:** 10.1371/journal.pmed.1003456

**Published:** 2020-12-02

**Authors:** Lucia Cilloni, Katharina Kranzer, Helen R. Stagg, Nimalan Arinaminpathy

**Affiliations:** 1 MRC Centre for Global Infectious Disease Analysis, School of Public Health, Imperial College London, London, United Kingdom; 2 Clinical Research Department, London School of Hygiene & Tropical Medicine, London, United Kingdom; 3 Biomedical Research and Training Institute, Harare, Zimbabwe; 4 Research Centre Borstel, Sülfeld, Germany; 5 Usher Institute, University of Edinburgh, Edinburgh, United Kingdom; UniversitatsKlinikum Heidelberg, GERMANY

## Abstract

**Background:**

Active case finding (ACF) may be valuable in tuberculosis (TB) control, but questions remain about its optimum implementation in different settings. For example, smear microscopy misses up to half of TB cases, yet is cheap and detects the most infectious TB cases. What, then, is the incremental value of using more sensitive and specific, yet more costly, tests such as Xpert MTB/RIF in ACF in a high-burden setting?

**Methods and findings:**

We constructed a dynamic transmission model of TB, calibrated to be consistent with an urban slum population in India. We applied this model to compare the potential cost and impact of 2 hypothetical approaches following initial symptom screening: (i) ‘moderate accuracy’ testing employing a microscopy-like test (i.e., lower cost but also lower accuracy) for bacteriological confirmation and (ii) ‘high accuracy’ testing employing an Xpert-like test (higher cost but also higher accuracy, while also detecting rifampicin resistance). Results suggest that ACF using a moderate-accuracy test could in fact cost more overall than using a high-accuracy test. Under an illustrative budget of US$20 million in a slum population of 2 million, high-accuracy testing would avert 1.14 (95% credible interval 0.75–1.99, with *p* = 0.28) cases relative to each case averted by moderate-accuracy testing. Test specificity is a key driver: High-accuracy testing would be significantly more impactful at the 5% significance level, as long as the high-accuracy test has specificity at least 3 percentage points greater than the moderate-accuracy test. Additional factors promoting the impact of high-accuracy testing are that (i) its ability to detect rifampicin resistance can lead to long-term cost savings in second-line treatment and (ii) its higher sensitivity contributes to the overall cases averted by ACF. Amongst the limitations of this study, our cost model has a narrow focus on the commodity costs of testing and treatment; our estimates should not be taken as indicative of the overall cost of ACF. There remains uncertainty about the true specificity of tests such as smear and Xpert-like tests in ACF, relating to the accuracy of the reference standard under such conditions.

**Conclusions:**

Our results suggest that cheaper diagnostics do not necessarily translate to less costly ACF, as any savings from the test cost can be strongly outweighed by factors including false-positive TB treatment, reduced sensitivity, and foregone savings in second-line treatment. In resource-limited settings, it is therefore important to take all of these factors into account when designing cost-effective strategies for ACF.

## Abstract 2

In this modeling study, Lucia Cilloni and colleagues simulate the cost and epidemiologic impact of active case-finding for tuberculosis in high-burden settings such as India.

## Author summary

**Why was this study done?:**

- Active case finding (ACF) is a strategy for reducing the burden of tuberculosis (TB) that aims to identify and treat TB cases in the community, as rapidly as possible.
- However, because ACF can be very resource-intensive, it is important to understand how best to conduct it, in the most cost-efficient way.
- Concentrating on the tests used to diagnose TB, we hypothesised that cheaper tests, although less accurate than more costly tests, would be important in bringing down the overall cost of ACF.

**What did the researchers do and find?:**

- Using a mathematical model of TB in urban slums in India, we simulated the cost and impact of an ACF intervention over the next 15 years.
- Contrary to our initial hypothesis, we found that the use of cheaper diagnostic tests with lower accuracy can in fact increase, not decrease, the overall cost of ACF.
- The reason is that the cost of a test can be outweighed by the costs of treatment, specifically false-positive (unnecessary) TB treatment, and second-line treatment for drug-resistant TB; cheaper tests would also have lower impact overall, as a result of their lower sensitivity.

**What do these findings mean?:**

- Our findings show how cheaper diagnostic tests may represent a false economy in ACF, i.e., leading to larger intervention costs overall, if they are also associated with reduced performance in ruling out TB.
- Those implementing ACF in resource-limited, high-burden settings should consider all of these factors in their planning, and not just the cost of the test.

## Introduction

Tuberculosis (TB) remains a major concern for global health, with 10.4 million incident cases globally in 2017 and approximately 1.3 million deaths [1]. The End TB Strategy calls for a reduction in incidence of 90% by 2035 [2]; to reach this target, it will be necessary to accelerate the rate at which TB cases are diagnosed and initiated on treatment [2]. Active case finding (ACF) is one way of doing so: Modelling suggests that ACF could be cost-effective under certain cost thresholds, particularly over longer (>10 years) time horizons [3] However, direct evidence for the potential impact of such measures in practice is limited [4], including in settings like India, the country with the world’s largest TB burden, which alone has an estimated 26% of the 3.6 million estimated ‘missing cases’ [1].

In response to these challenges, a key component in India’s 2017 National Strategic Plan for TB Elimination is the need to perform intensive, sustained ACF in specific populations such as urban slums, which are known to have a greater TB burden than the general population [5]. In general, ACF in these and other settings employs screening for symptoms suggestive of TB, followed by bacteriological testing. The latter is often performed using molecular tools such as Xpert MTB/RIF (hereafter referred to as ‘Xpert’), a cartridge-based nucleic acid amplification test. More recently developed, the ‘Ultra’ diagnostic platform offers higher sensitivity than Xpert, but at the expense of lower specificity [6]. In the present work, we concentrate on the deployment of Xpert. Key advantages of Xpert are that (i) it is more sensitive than the conventionally used smear microscopy, which can miss up to half of pulmonary TB cases [7] and (ii) it offers detection of rifampicin resistance at the point of TB diagnosis [1,8], thus allowing the timely initiation of second-line treatment. However, those cases detectable through smear microscopy tend to be the most infectious [9], and smear microscopy is considerably cheaper than Xpert. Therefore, the use of smear microscopy may achieve substantial epidemiological impact, at a lower cost.

Could smear-based ACF be more cost-effective than Xpert-based ACF, in settings such as India? What are the benefits of using Xpert in comparison to smear microscopy, and vice versa? Our scope is not simply yield (defined as the proportion of positive cases identified), a major focus of operational research, but also potential epidemiological impact, taking into account the potential transmission implications of ACF. In line with India’s National Strategic Plan, we model case-finding efforts in conditions typical of urban slums using a transmission model combined with a simple costing approach. Building on previous modelling analyses of ACF [10,11], in this work we focus on both the specificity and sensitivity of the diagnostic strategy employed in ACF.

## Methods

### Transmission model

**Fig 1:**
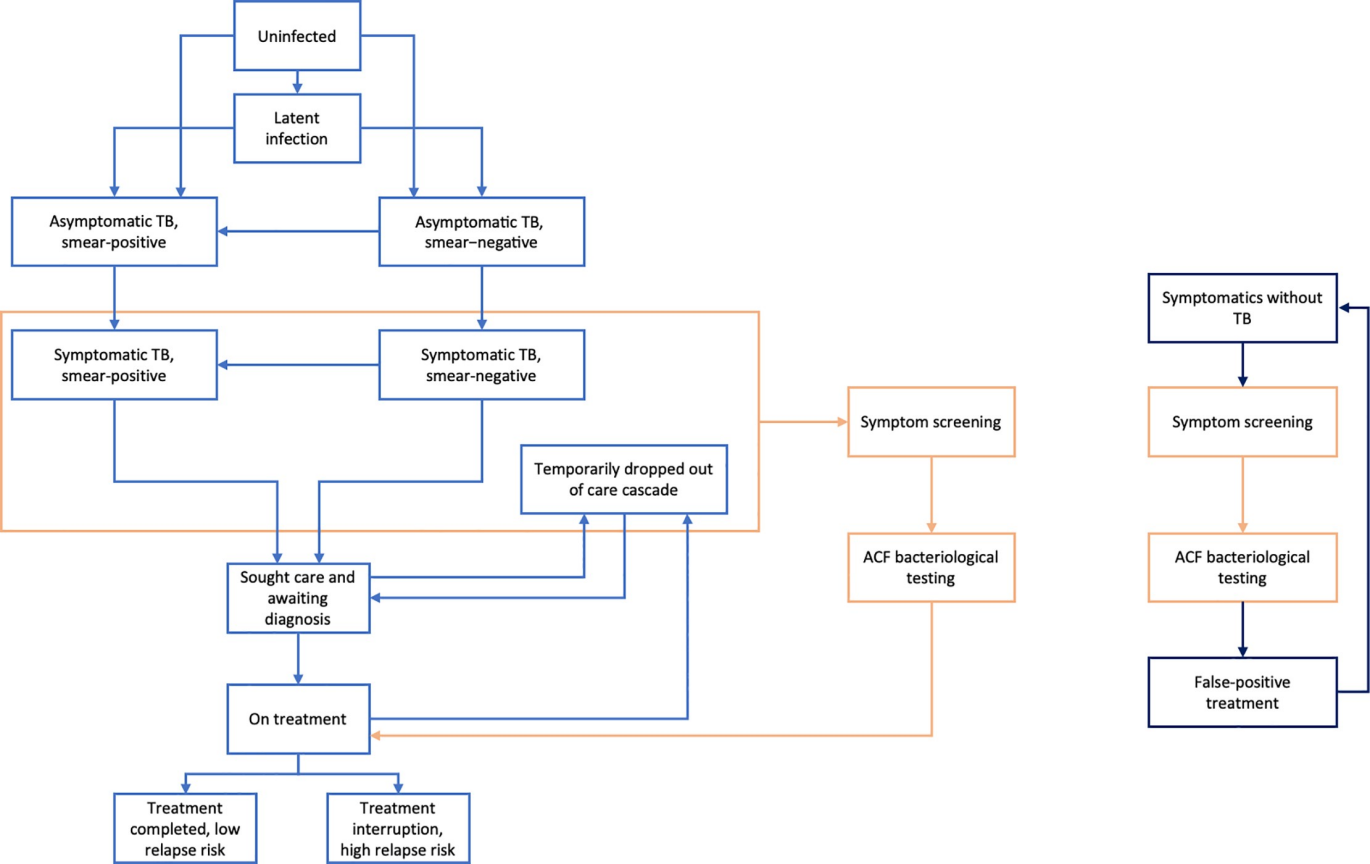
Schematic illustration of the model. The tuberculosis (TB) transmission model distinguishes TB by smear status and by symptom status. Upon developing symptoms, symptomatic individuals seek care through either the private or public sectors (‘passive’ TB services) after a certain delay, estimated to match data in Table 2. Although not shown here for clarity, the model captures these sectors separately, including the lower standard of TB care in the private sector (see S1 Text for full model details). Those successfully diagnosed initiate TB treatment; we assume that 15% of diagnoses in the public sector are conducted with Xpert, the remainder by microscopy. All those lost from the TB care cascade, whether because of missed diagnosis, pre-treatment loss to follow-up, or failed treatment, temporarily disengage from care-seeking, before once again seeking care after a given delay. Compartments shown in orange denote the effect of an active case finding (ACF) intervention on this ‘passive’ system; we assume that ACF consists of initial symptom screening, followed by microbiological confirmation. Meanwhile, the separate compartments on the right represent a subset of the general population that may be detected by the ACF intervention (represented by the orange compartments) because they have TB-like symptoms, but without TB: These may include, for example, individuals with chronic obstructive pulmonary disease, bronchitis, and other lung conditions. They incur a cost to the health system for diagnosis and—if they are mistakenly diagnosed with TB—a cost in false-positive treatment. The number incorrectly identified as having TB is dependent on the specificity of both the screening and confirmatory stages. Finally, at any stage individuals may die of natural causes or of TB (in the diseased compartments) or recover spontaneously. For simplicity, these transitions are not shown in the figure (see S1 Text for full model details).

We developed a dynamic transmission model of TB, illustrated in Fig 1 and described in further technical detail in S1 Text. The model captures key features in the natural history of TB, including asymptomatic but infectious disease, smear status, TB mortality, and spontaneous cure. The model focuses on pulmonary TB and disregards extrapulmonary TB, assuming ACF to focus on the former. Amongst those with TB (indicated by the orange box in Fig 1), we assumed that smear-negative pulmonary TB cases are, on average, 20% as infectious as smear-positive ones [9] (see Table 1 for model parameters). We incorporated ‘passive’ TB service delivery, including the split between public and private sectors that is a major feature of the health system in India [12]. Additionally, the model also distinguishes drug-susceptible TB (DS-TB) and drug-resistant TB (DR-TB), the latter including both rifampicin-resistant and multi-drug-resistant forms of TB. Although DR-TB only accounts for approximately 5% of the TB burden in India, it also consumes a disproportionate amount of the TB budget due to the high cost of second-line treatment [1]. In the model, we account for the delay in recognition of DR-TB as a result of missed opportunities for drug sensitivity testing, as well as the potential impact of Xpert-based ACF in reducing this delay.

**Table 1:** List of model parameters.

Parameter			Symbol	Value (95% credible interval)	Source/notes
*Natural history*					
Infection rate, smear-positive DS-TB			β_DS_	9.10 years^−1^ (7.35–10.70)	Fitted to epidemiological data (Table 2)
Infection rate, smear-positive DR-TB			β_MDR_	5.19 years^−1^ (4.12–6.26)	
Relative infectiousness, smear-negative versus smear-positive			ε	0.2 (0.1–0.3)	[9]
Rate of progression to active disease from latency			*a*	0.0005–0.0015	[38]
Proportion of infections being ‘fast’ progressors to active disease			*p*_Fast_	0.05–0.15	[39]
Per capita rate of initial care-seeking upon first developing symptoms			*r*_CS_	0.73 years^−1^ (0.57–0.91)	Fitted: corresponds to a mean initial delay of over a year
Per capita rate of repeat care-seeking			$r_{cs}^{\left(2\right)}$	12 years^−1^ (9–15)	Assumption: corresponds to a mean delay of 1–6 weeks
Per capita rate of smear conversion		Symptomatic TB	*m*_0_	0.71 years^−1^ (0.40–1.04)	Fitted to prevalence survey data (Table 2)
		Asymptomatic TB	*m*_1_	0.63 years^−1^ (0.62–0.64)	
Per capita rate of symptom development		Smear-positive TB	*e*_0_	1.24 years^−1^ (1.02–1.65)	
		Smear-negative TB	*e*_1_	2.37 years^−1^ (1.90–3.05)	
Proportion of prevalent TB cases that are smear-positive			ω_+_	0.6 (0.5–0.7)	[1]
Per capita rate of relapse		After treatment completion	*r*_1_	0.032 years^−1^ (0.024–0.04)	[38]
		After treatment default	*r*_2_	0.14 years^−1^ (0.105–0.175)	
		Long-term (>2 years) relapse risk	*r*_3_	0.002 years^−1^ (0.0011–0.0019)	
Per capita rate of spontaneous recovery			γ	0.1667 years^−1^ (0.1250–0.2083)	[38]: together, yielding a 50% case fatality rate over 3 years of untreated TB
Per capita rate of mortality, untreated TB			μ_TB_	0.1667 years^−1^ (0.1250–0.2083)	
Proportion reduction in susceptibility to reinfection owing to previous infection			ρ	0.21 (0.15–0.25)	[40]
Per capita background mortality rate			μ	0.0152 years^−1^	[38]: corresponds to a mean life expectancy of 66 years
Per capita birth rate			*b*	0.0682	[41]: adjusted to yield 2.4% annual population growth from 1970
*Diagnosis (routine TB services*, *in absence of ACF)*					
Proportion seeking care from private sector			*p*_1_	0.5 (0.4–0.6)	Assumption, consistent with [12]
Proportion correctly diagnosed per provider visit		Public sector	$p_{0}^{\left(Dx\right)}$	0.83 (0.81–0.85)	[42]
		Private sector	$p_{1}^{\left(Dx\right)}$	0.7 (0.6–0.8)	Assumption
Proportion of diagnoses successfully initiating treatment		First-line, public sector	$p_{0}^{\left(Tx\right)}$	0.88 (0.85–0.91)	Aggregated for first- and second-line [42]
		First-line, private sector	$p_{1}^{\left(Tx\right)}$	0.7 (0.6–0.8)	Assumption
		Second-line, public only	$p_{0}^{\left(Tx2\right)}$	0.88 (0.85–0.91)	Aggregated for first- and second-line [42]
Proportion of TB recognised as DR-TB at point of diagnosis (public only*)			*p*_DST_	0.12 (0.08–0.20)	[22]
*Treatment*					
Per capita rate of regimen completion		First-line	*d*_TxFL_	2 years^−1^	[1,3]: corresponds to a duration of 6 months
		Second-line	*d*_TxSL_	0.5 years^−1^	[1]: corresponds to a duration of 2 years
Proportion first-line treatment success		Public sector	$c_{0}^{1}$	0.85 (0.83–0.87)	[1,3]
		Private sector	$c_{1}^{1}$	0.6 (0.5–0.7)	Assumption
Proportion second-line treatment success (public only*)			*c*_2_	0.46 (0.44–0.5)	[1,3]
Amongst DR-TB cases failing first-line treatment, proportion successfully transferred onto second-line treatment (public only*)			*p*_SL_	0.88 (0.85–0.92)	Assumption
Rate of DR-TB acquisition amongst DS-TB cases on first-line treatment			*r*_MDR_	0.01 years^−1^	[1,38]
*Active case finding*					
High-accuracy test performance (consistent with available data for Xpert)	Sensitivity (smear-positive TB)		*s*_1_	1	Assumption (at least as sensitive as smear)
	Sensitivity (smear-negative TB)		*s*_0_	0.7 (0.6–0.8)	[43,44]
	Specificity		σ	0.99 (0.90–1.0)	[45]**
	Per capita rate of performing diagnostic test		*d*_Dx_	52 years^−1^	We assume 1 week for sample collection, transportation, and analysis
	Proportion of TB recognised as DR-TB at point of diagnosis		*p*_DSTA_	0.95 (0.90–0.97)	[8]
Moderate-accuracy test performance (consistent with available data for smear)	Sensitivity (smear-positive TB)		*s*_1_	1	Simplifying model assumptions
	Sensitivity (smear-negative TB)		*s*_0_	0	
	Specificity		*σ*	0.98 (0.93–1.0)	[45]**
	Per capita rate of performing diagnostic test		*d*_Dx_	52 years^−1^	We assume 1 week for sample collection, transportation, and analysis
Symptom screening (any TB symptom)	Sensitivity		*s*	0.70 (0.58–0.82)	[14]
	Specificity***		σ	0.61 (0.35–0.87)	[14]
	Per capita rate of performing symptom screening		***d***_**sx**_	365 years^−1^	Assumption: corresponds to 1 day

*We assume that all DR-TB management occurs in the public, not private, sector.

**In the parameter sampling, we adopt only those joint parameter sets in which Xpert specificity is greater than that of smear.

***The size of the non-TB symptomatic (NTS) population was calculated using the specificity of the symptom screening method used (see S1 Text for full model specifications). For a strategy screening for ‘any TB symptom’, the size of the NTS population would therefore be 39% of the size of the population in which TB dynamics are modelled.

ACF, active case finding; DR-TB, drug-resistant tuberculosis; DS-TB, drug-susceptible tuberculosis; TB, tuberculosis.

**Table 2:** Data used to calibrate the compartmental model.

Data		Calibration target (95% uncertainty interval)	Source/notes
Slum prevalence (per 100,000 population) of culture-positive TB, as of 2012		432 (341–527)	Drawn from [5], using prevalence of culture-positive TB (259 per 100,000), and inferring the slum prevalence from the univariate odds ratio of culture-positive TB in slum versus non-slum settings (2.3), together with an assumed slum size of 20% of the urban population
Slum ARTI, as of 2006		2.5% (1.9%–3.1%)	[46] and Chadha VK. Personal communication. “Unpublished data, First round of zonal ARTI surveys in India, 2000–2003”
Proportion of TB incidence that is DR-TB as of 2018		5% (4%–6%)	[47]
Proportion of prevalent TB having any TB symptoms		70% (58%–82%)	By definition, same as assumed value of sensitivity of symptom screening (Table 1)
Proportion of prevalent TB that is smear-positive as of 2012	In symptomatic individuals	67% (60%–74%)	[5]
	In asymptomatic individuals	66% (56%–77%)	[5]

DR-TB includes both rifampicin-resistant and multi-drug-resistant forms of TB. Although largely drawn from a prevalence survey in Chennai, South India [5], these data are broadly consistent with prevalence surveys in urban settings elsewhere in India [48].

ARTI, annual risk of tuberculosis infection; DR-TB, drug-resistant tuberculosis; TB, tuberculosis.

We separately simulated the dynamics of a non-TB symptomatic (NTS) population, i.e., individuals who would be eligible for a TB diagnosis based on the presence of TB-like symptoms, but who do not have TB. This population is necessary for tracking the overall number of diagnostic tests being conducted during the case-finding intervention, as well as the unnecessary treatment of false-positive TB [13].

### Model calibration

The model described above was calibrated to annual risk of *Mycobacterium tuberculosis* infection and prevalence estimates representative of urban slums in India (Table 2). To inform model parameters for symptom onset from asymptomatic stages, and for progression of smear status with developing disease, we drew from a prevalence survey in Chennai [5]. In particular, we calibrated the model to the proportion of prevalent TB cases that are symptomatic and the proportion smear-positive amongst those who had not yet presented for care, stratified by symptom status (Table 2).

Uncertainty was estimated using Latin hypercube sampling to sample model parameters from their respective ranges (10,000 samples) and simulating the model to 2018, as described in S1 Text. Parameter sets were accepted in the sampling if they provided model projections that fell within the calibration target ranges, while the rest were rejected.

### Intervention

Using the calibrated model, we simulated an ACF algorithm using symptom screening followed by a confirmatory microbiological test. We assumed that all TB diagnoses through ACF would be microbiologically confirmed, i.e., we assumed that there is no role for presumptive clinical diagnosis in ACF. For the screening stage, we modelled the use of symptom screening, with individuals reporting any symptom suggestive of TB deemed eligible for microbiological confirmation. For the sensitivity and specificity of this approach for TB, we drew from a recent meta-analysis of screening approaches [14]. We also performed sensitivity analyses (described below) under an alternative screening approach: identifying those with prolonged cough, rather than any TB symptom (with prolonged cough screening having higher specificity but lower sensitivity than ‘any TB symptom’).

For the confirmatory test, we distinguished 2 types of strategy: a ‘moderate accuracy’ strategy, using a confirmatory test with performance and cost consistent with smear microscopy, and a ‘high accuracy’ strategy, using a confirmatory test with performance and cost consistent with Xpert (see Table 1 for parameters). The reason for these designations is that—although there exists data from clinical trials on the performance of smear microscopy and Xpert—there is currently little evidence for characteristics such as the specificity of either test in real-world conditions. In particular, test specificity is often assessed against culture as a reference standard and thus does not address how to interpret smear-positive, culture-negative cases that nonetheless show clear signs of having TB [15,16]. We do not address the important question of how reference standards might be improved to meet these challenges. Instead, our analysis casts light on which types of uncertainty are most critical for future studies to address in strategic planning for ACF activities. Consistent with the capabilities of smear microscopy and molecular tests such as Xpert, we assumed that the high-accuracy test can detect rifampicin resistance at the same time as detecting TB, while the moderate-accuracy test cannot. We assumed that all individuals recognised as having rifampicin resistance are immediately initiated on second-line therapy, while those with unrecognised rifampicin resistance are only switched to second-line therapy after failing first-line treatment.

We considered ‘sensitivity’ as the proportion of TB cases that yield a positive microbiological result under a given test, and likewise ‘specificity’ as the proportion of individuals without TB who yield a negative microbiological result. Moreover, we assumed that all smear-positive cases test positive through a smear-like test, and that no smear-negative cases do (i.e., sensitivities of 1 and 0, respectively). This is a simplification for model purposes: We also performed a sensitivity analysis under alternative assumptions, where smear status is associated with a given probability of diagnosis.

We simulated the intervention at different levels of coverage to run from 2020 to 2035, scaled up linearly over the first 4 years (2020–2023), ultimately to screen a given proportion of the population per year. We assumed for simplicity that this proportion is selected at random from the slum population each year. The impact of ACF was measured as the percentage reduction in cumulative incidence between 2020 and 2035. Finally, we calculated the incremental costs of the intervention relative to a baseline of status quo, i.e., current standards of TB care continued indefinitely, without ACF.

### Economic evaluation

We focus on the programmatic perspective, i.e., considering only costs borne by the TB programme, and ignoring broader societal costs. The model accounts for ‘passive’ TB services, i.e., those routine services (diagnosis and treatment, both first- and second-line) independent of ACF, and contingent on patients presenting for care. Since these services could be lowered by the rollout of ACF, this will impact the overall incremental costs. Concentrating on programmatic costs, we counted only the costs of diagnosis and treatment in the public sector, and not the private sector. Moreover, new second-line regimens introduced in 2017 present a range of possible costs for DR-TB treatment. However, as the focus of the current study is on ACF, for simplicity we assumed costs and outcomes consistent with currently used regimens (Tables 1 and 3), while performing sensitivity analyses with respect to this assumption. For all unit costs, for simplicity we assumed uncertainty intervals of ±20%.

**Table 3:** Unit (service) costs used in the analysis.

Unit cost	Cost in US dollars (95% uncertainty interval)	Source/notes
*Active case finding*		
Symptom screening	2.00 (1.60–2.40)	Table S15 from [49]—unit cost of symptom screening in South Africa.
Sputum smear microscopy	2.26 (1.81–2.71)	Table 2 from [50] reports a unit cost of US$1.13 for a single acid-fast bacillus smear in India. With a minimum of 2 smears required for diagnosis, we double this cost.
Xpert MTB/RIF	17.53 (14.02–21.04)	Table S12 from [49]—difference between unit cost of Xpert and microscopy is given to be US$16.4.
*Treatment (cost per patient-month)*		
First-line treatment	2.42 (1.93–2.90)	For an average total cost of US$14.5 (US$11.6–US$17.4). Annex 4 from [28]—provider drug costs for DS-TB in India.
Second-line treatment	100 (80–120)	For an average total cost of US$2,400 (US$1,920–US$2,880) for the full regimen [28,51].

For simplicity we ignore the ‘new’ second-line regimens, as it is unclear what proportions of patients will be eligible for the different treatment options. However, in S1 Text we provide a sensitivity analysis with respect to the potential future uptake of these regimens. To capture uncertainty in costs, we allowed variation by ±20% for each of these cost components.

DR-TB, drug-resistant tuberculosis; TB, tuberculosis.

For the ACF costs, we assumed that all patients identified through ACF are linked to treatment in the public sector. We focused for simplicity on service costs related to the numbers of confirmatory tests and patient-months of first- and second-line treatment incurred by the intervention. In doing so, we are neglecting the ‘intervention costs’ needed to facilitate the delivery of these interventions (e.g., human resources). As discussed below, these are important cost components in any exercise aiming to estimate the full costs of ACF [17]. For the purpose of the present study, however, if these intervention costs are similar for moderate- and high-accuracy testing, they may not substantially impact a comparison in cost between the two.

Finally, we tracked the costs of treating false-positive TB diagnoses, as driven by the imperfect specificity of the screening and diagnostic tools involved, and the low prevalence of TB (<5% amongst those with symptoms, even in urban slums). Unnecessary TB treatment carries heavy societal costs, including avoidable stigma [13], as well as the monetary costs to patients and households (e.g., travel costs) involved in completing a regimen of TB treatment [18]. Such factors are outside the scope of our current study: Here we focus on unnecessary programmatic spending on the treatment of false-positive TB, recognising (as discussed below) that this approach represents only one narrow part of the overall adverse effects of false-positive treatment [13]. All unit costs are provided in Table 3. Overall, we caution that our estimates should not be interpreted as representing the overall cost of any ACF intervention, given that they miss these cost components, as well as the implementation costs described above.

### Sensitivity analysis

To test model sensitivity to alternative screening strategies, we first simulated the impact on incidence and incremental costs of using prolonged cough for symptom screening, which has higher specificity and lower sensitivity than the symptom screening strategy used in the main analysis, any symptom suggestive of TB. For additional sensitivity analyses, as a focal model output, we calculated the cumulative cases averted between 2020 and 2035 under an assumed budget of US$20 million, calculating the ratio of cases averted between the high-accuracy and moderate accuracy scenarios. This ratio offers an estimate of the relative cost efficiency of the 2 approaches. We examined model sensitivity to individual parameters by conducting a partial rank correlation between this model output and each of the model inputs listed in Table 1. We additionally evaluated the focal model output under different scenarios: (i) assuming that smear microscopy can detect 25% and 75% of smear-negative and -positive cases, respectively (as opposed to 0% and 100% in the main analysis) and (ii) using alternative scenarios for the burden and management of DR-TB, which can consume a disproportionate share of programmatic spending. We modelled a scenario consistent with Mumbai, which has a substantially higher DR-TB burden than the national average. We also modelled a scenario with the adoption of new, more effective, and less costly second-line regimens.

### Planning and execution of methods

At the outset of this analysis, the modelling plan focused on the confirmatory test, assuming the use of ‘any symptom suggestive of TB’ as a screening strategy. Subsequently, our main adjustment to this initial plan was in response to constructive reviewer comments, on the need to better understand the influence of the screening algorithm. In response, we incorporated the additional analysis as described above, on the alternative use of prolonged cough as a screening strategy. Also in response to reviewer comments, we incorporated the additional sensitivity analyses described above, for the alternative scenarios for the burden and management of DR-TB. Our initial modelling plan otherwise underwent no data-driven changes.

## Results

**Fig 2:**
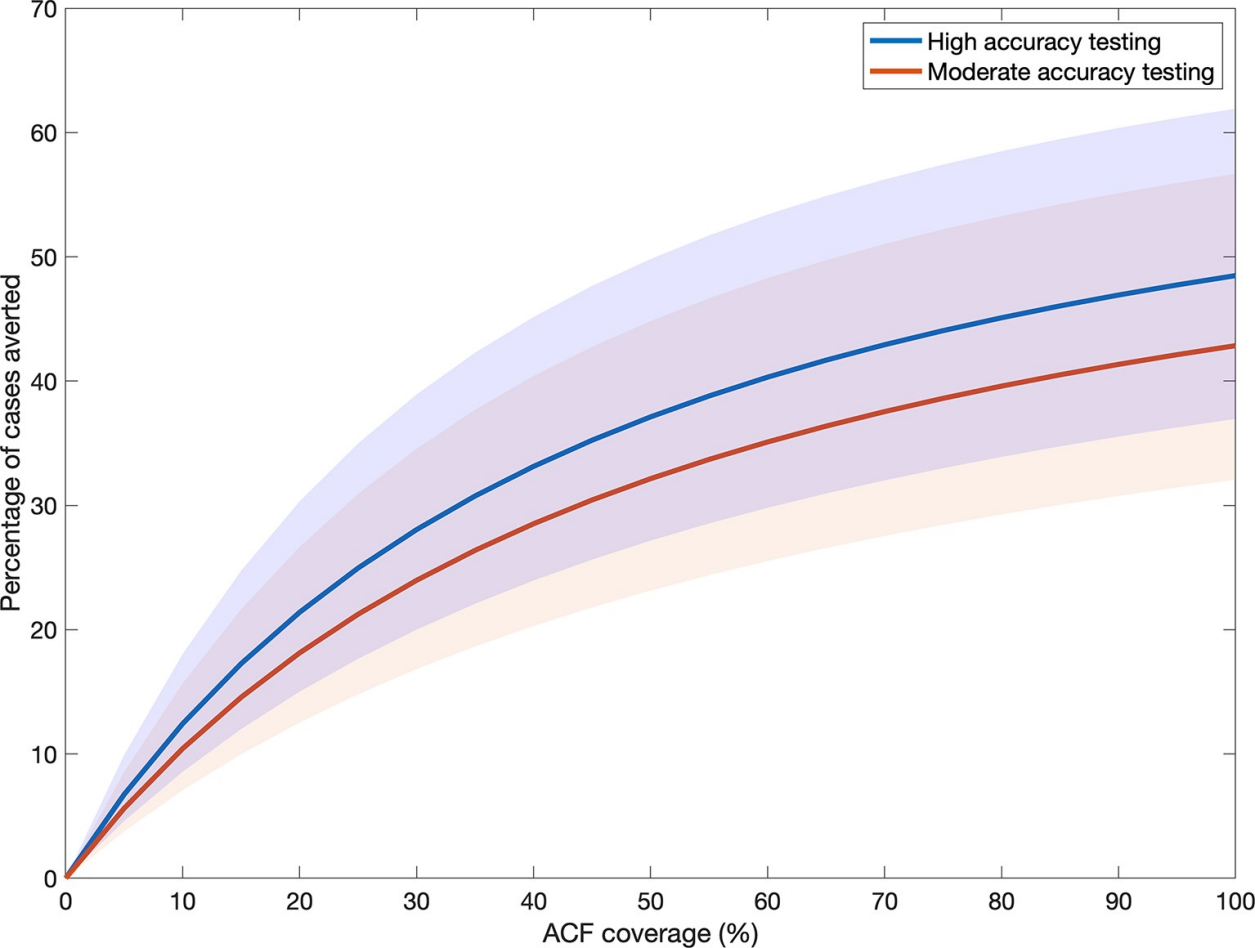
Active case finding (ACF) impact as a function of coverage. Here we measure impact as the percentage reduction in cumulative incidence between 2020 and 2035, and coverage as the proportion of the slum population being screened per year. We assume for simplicity that a randomly selected proportion of the slum population is selected for screening each year, independent of screening in previous years. An ACF intervention with symptom screening is followed by bacteriological confirmation, using either a smear-like test (red curve) or an Xpert-like test (blue curve). The shaded areas represent the 95% credible intervals. Each of the curves is generated by taking a range of annual screening proportion from 0 (no ACF) to 1 (whole slum population screened for symptoms once a year): The upper endpoints of each curve occur at the upper limit of this range. Overlap between these areas does not imply a lack of significant difference between the interventions, as points in the red and blue areas are correlated. Indeed, the relative impact of the 2 strategies is robust to this parameter uncertainty (see main text).

Results of model calibration are shown in Fig A in S1 Text. Simulating the ACF interventions, Fig 2 shows model projections for how epidemiological impact varies with coverage (proportion of the slum population screened per year). The higher the proportion of the slum population being screened each year, the greater the reduction in the prevalent pool of infectious TB; the impact shown in Fig 2 is the result of reducing opportunities for transmission in this way, measuring impact as the percentage reduction in cumulative incidence between 2020 and 2035. The figure illustrates that high-accuracy testing would have a greater impact than a moderate-accuracy strategy, at a given level of coverage, as a result of its higher sensitivity. Even with the latter diagnosing the most infectious cases, this difference in impact is robust to parameter uncertainty. Taking the example of 50% coverage, the relative impact of the high- versus moderate-accuracy strategies is 1.16 (95% credible interval 1.11–1.22).

**Fig 3:**
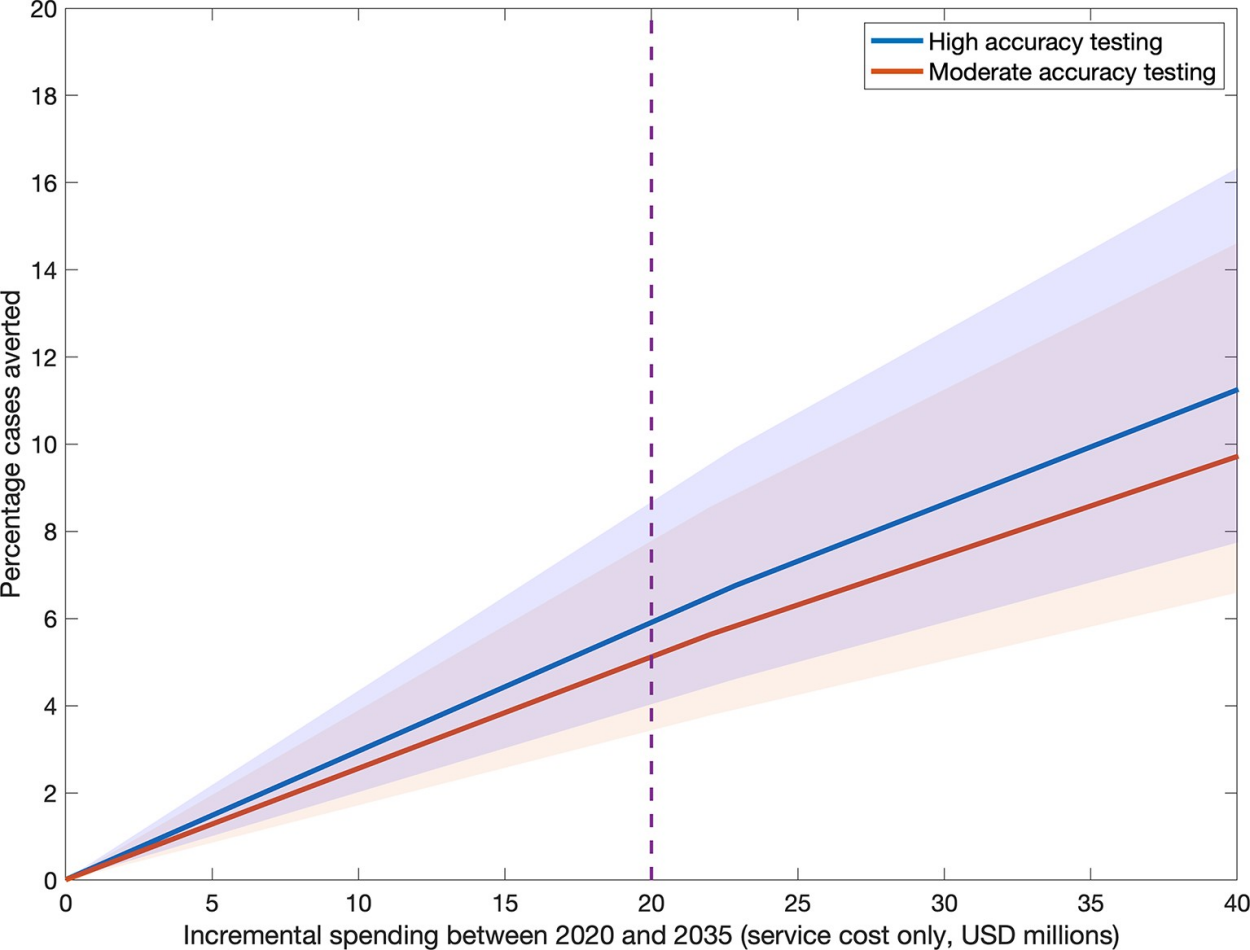
Active case finding (ACF) impact as a function of incremental programmatic spending, in an assumed slum population of 2 million people. As in Fig 2, we measure impact as the percent cases averted by ACF. Incremental spending (in millions of US dollars [USD]) is the overall service cost of diagnostics and treatment, relative to a baseline scenario of no ACF, and assuming current conditions continue indefinitely. The vertical dashed line shows an illustrative budget of US$20 million; in spite of using a lower-cost test, a moderate-accuracy strategy is overall less cost-efficient than a high-accuracy one. The shaded areas represent the 95% credible intervals.

Fig 3 illustrates how impact varies with incremental spending between 2020 and 2035 under the 2 ACF strategies. Here we assume an illustrative slum population of 2 million people, based on the mean population of major cities in India of approximately 10 million, of whom roughly a third live in slums [19] (for smaller or larger cities, incremental spending will be proportional to population size). The vertical dashed line shows the percent of cases that could be averted under an illustrative spend of US$20 million. As noted above, this spend relates only to service costs of ACF, and does not reflect the full implementation costs of ACF. Under this budget, the impact of high-accuracy testing, relative to that of moderate-accuracy testing, is 1.14 (95% credible interval from simulation 0.75–1.99). Overall, therefore, results suggest that high-accuracy testing would not only be more impactful at a given level of coverage (Fig 2), it could—under certain circumstances—also be more cost-efficient.

**Fig 4:**
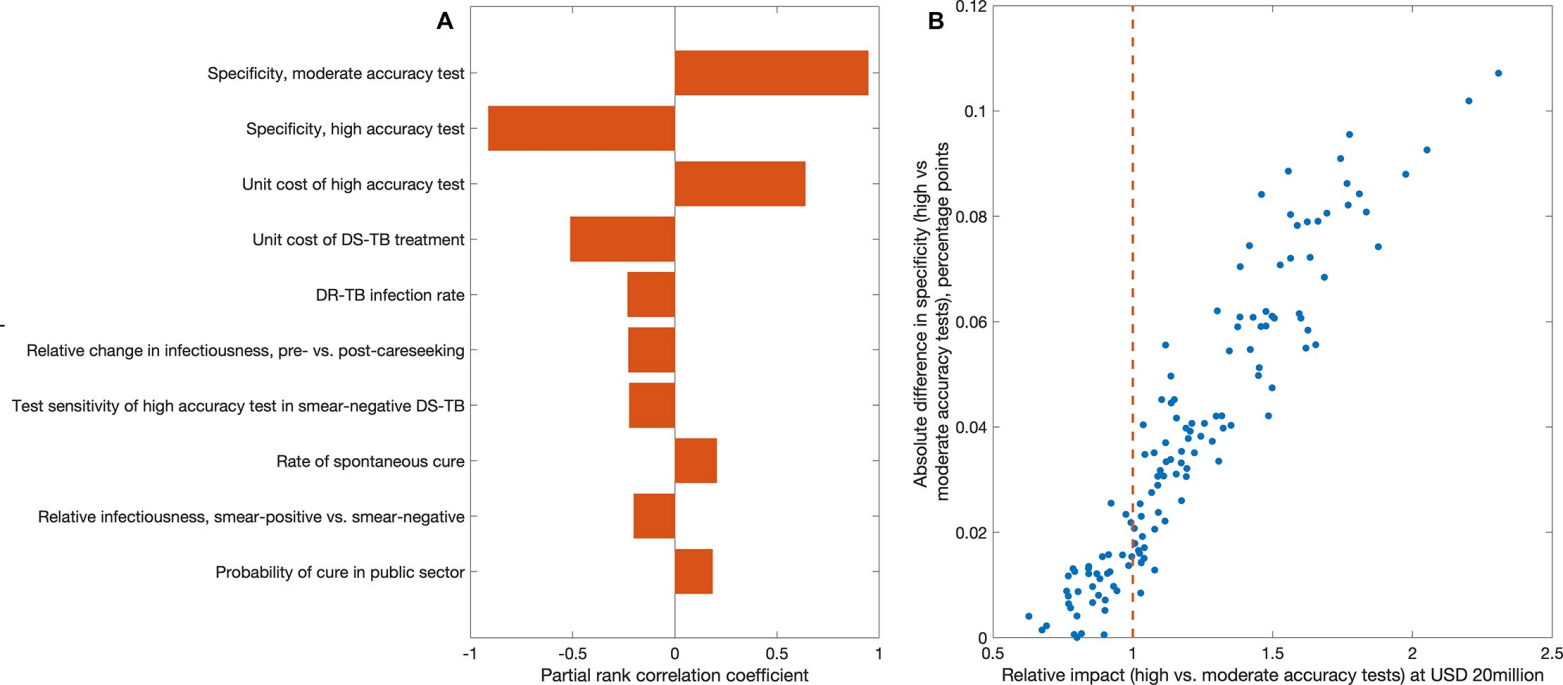
Sensitivity analysis to identify key model parameters in the relative impact of high- versus moderate-accuracy strategies, under a given budget of US$20 million between 2020 and 2035. Here, we define relative impact as the ratio of the number of cases averted over this period by a high-accuracy testing strategy relative to a moderate-accuracy one. In Fig 3, this focal model output is estimated to be 1.14 (95% credible interval from simulation 0.75–1.99). (A) Partial rank correlation coefficients of model parameters against relative impact, showing only the 10 highest correlations, and highlighting the test specificities as being the 2 most influential parameters. (B) Association between relative impact and test specificity, showing that rather than individual specificities, it is their absolute difference that matters most for relative impact. All points to the right of the vertical dashed line correspond to a high-accuracy test being more impactful than a moderate-accuracy one; these results suggest that an absolute specificity difference of at least 3 percentage points is sufficient to ensure that a high-accuracy test is more impactful than a moderate-accuracy one. DR-TB, drug-resistant tuberculosis; DS-TB, drug-susceptible tuberculosis; USD, US dollars.

Fig 4 shows analysis to better resolve these circumstances. Fig 4A shows the most influential model parameters in the relative impact of a high- versus moderate-accuracy test at a budget of US$20 million, highlighting the respective specificities of the 2 tests as the most influential parameters. In particular, the difference in specificity between the 2 tests is an important driver: Fig 4B shows the absolute difference, plotted against relative impact, highlighting that a high-accuracy test would be robustly more cost-efficient than a moderate-accuracy one, as long as it has specificity that is at least 3 percentage points greater.

**Fig 5:**
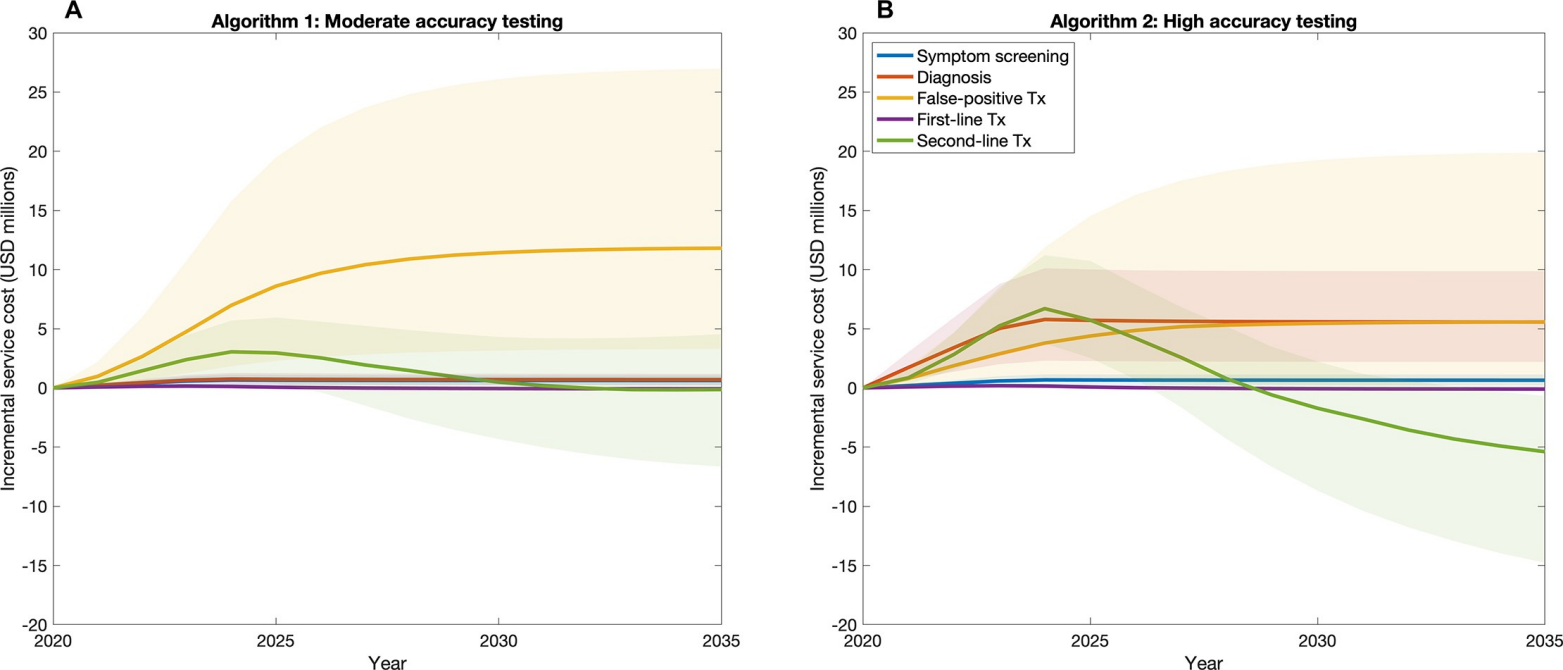
Breakdown of the incremental ACF service cost, shown here at 50% screening coverage for the 2 ACF algorithms. (A) Moderate-accuracy testing. The major driver of the incremental cost of moderate-accuracy testing is the treatment of false-positive TB in the non-TB symptomatic population (orange line). (B) High-accuracy testing. Notably, the cost of treatment of false-positive individuals is nearly halved when using an Xpert-like test for diagnosis (orange line). False-positive TB treatment costs and diagnosis costs (red line) are the 2 components that are the main cost drivers for high-accuracy testing. The shaded areas represent the 95% credible intervals. ACF, active case finding; TB, tuberculosis; Tx, treatment; USD, US dollars.

Fig 5 illustrates why specificity is a driving factor, as well as identifying additional drivers in the impact of a high-accuracy test. Taking the example of 50% coverage, the figure shows the separate components of incremental cost through time for both diagnostic tests. Fig 5A illustrates that false-positive treatment is by far the largest cost driver of a moderate-accuracy ACF intervention, followed by the cost of second-line treatment. For a high-accuracy test (Fig 5B), major cost drivers are the cost of the test itself, the cost of treating false-positive diagnoses, and the cost of second-line treatment. Two comparisons bear mention. First, the rate of false-positive TB diagnosis is strongly affected by the specificity of the confirmatory test, with a moderate-accuracy test being associated with 350,000 false-positive treatment initiations (95% credible interval 100,000–710,000), compared to 180,000 for high-accuracy testing (95% credible interval 30,000–560,000). Second, the effects of each test on DR-TB incidence plays an important role in cost dynamics: By identifying DR-TB cases early, high-accuracy testing tends to have a stronger impact on reducing DR-TB incidence, thus leading to cost savings in second-line treatment costs, over 15 years.

**Fig 6:**
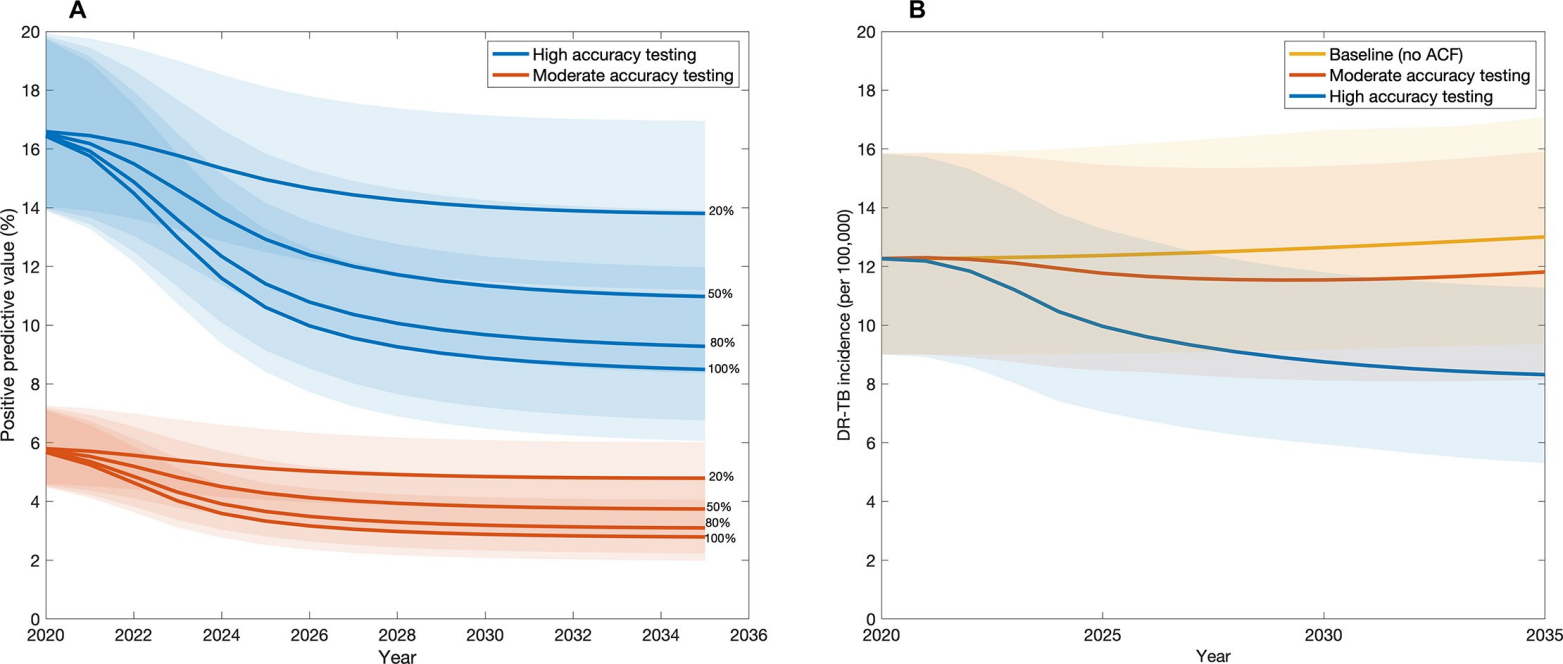
Additional comparisons between testing strategies. (A) Comparison of the positive predictive value (PPV) of active case finding (ACF) strategies. The panel shows the PPV of the entire diagnostic algorithm (including symptom screening), and not just that of the confirmatory test. Percentages on the right-hand side of the figure (20%, 50%, etc.) show ACF coverage scenarios, i.e., the proportion of the slum population being screened per year. The shaded areas represent the 95% credible intervals. Overall, as tuberculosis (TB) prevalence is reduced over time by ACF, the PPV also decreases. Improved diagnostic algorithms, with improved specificity, may be needed in these advanced stages. (B) Comparison of both testing strategies shown in Fig 3, by their impact on the incidence of drug-resistant TB (DR-TB) over time, at fixed 50% coverage. Because a high-accuracy test is able to diagnose rifampicin resistance at the point of TB diagnosis, it can contribute strongly to long-term reductions in DR-TB incidence, thus also averting future costs of second-line treatment (Fig 5B). A moderate-accuracy strategy also leads to a decline in DR-TB incidence, although to a lesser extent, as individuals with DR-TB are only switched to second-line therapy after failing first-line therapy.

Cost drivers are further explored in Fig 6. The role of false-positive TB treatment is illustrated in Fig 6A, which shows how the positive predictive value of the whole diagnostic algorithm (including symptom screening) changes over time under both algorithms. For both scenarios, this value substantially decreases over time as a result of decreasing TB prevalence in the community but remains considerably lower for smear microscopy than for Xpert. Likewise, the role of DR-TB is further illustrated in Fig 6B, which shows the strong DR-TB incidence reductions that would be brought about by a high-accuracy testing strategy over time.

The cost efficiency of the overall ACF algorithm can be shaped as much by the choice of screening algorithm as by the choice of confirmatory test. Fig B in S1 Text shows results for incidence and impact under an alternative screening algorithm, using prolonged cough as the eligibility criterion for confirmatory testing, an approach having lower sensitivity and higher specificity (25% and 96%, respectively [14]). At a given level of coverage, such a screening approach would reduce the overall impact of both high- and moderate-accuracy strategies, owing to its lowered sensitivity. As a result of its higher specificity, such an approach also substantially reduces the number of false-positive treatments overall, narrowing this specific advantage of a high-accuracy test. However, under a given budget of US$20 million (as used in Fig 4), a prolonged cough screening algorithm could allow many more people to be screened over time, potentially leading to a greater impact overall (Table A and Fig B in S1 Text) than that shown in Fig 3. In turn, this enhanced impact promotes the long-term effect of the high-accuracy test in averting second-line costs. The overall effect is for the relative impact of the 2 testing strategies at a budget of US$20 million, to remain qualitatively similar to that estimated from Fig 3 above, and indeed shifted in favour of the high-accuracy test.

Finally, we conducted sensitivity analyses. Focusing on the relative impact of the high- versus moderate-accuracy strategies shown in Fig 3, Fig D in S1 Text shows how this focal model output varies under a range of scenarios. The figure illustrates that the essential qualitative results remain under alternative scenarios for the sensitivity of smear microscopy, the burden and management of DR-TB, and the size of the NTS population.

## Discussion

ACF is potentially highly impactful, but also highly resource-intensive: Our analysis therefore addresses the critical need to optimise its cost-effectiveness. We used mathematical modelling to examine strategies for microbiological testing in ACF for TB, aiming to identify the type of confirmatory test that would yield the greatest epidemiological impact at the lowest cost.

## Findings

We hypothesised that a moderate-accuracy testing strategy, using a relatively cheap test that can nonetheless detect the most infectious cases, may be more cost-efficient (achieving incidence reductions at lower cost) than a high-accuracy strategy. However, our results suggest that the converse is true (Fig 3) wherever lower test cost is associated with reduced specificity. The reasons are 3-fold: (i) small improvements in test specificity can translate to large reductions in unnecessary (false-positive) TB treatments, which outweigh the cost of the test; (ii) a high-accuracy test that can also identify rifampicin resistance can offer long-term savings by reducing the burden of rifampicin-resistant TB (and thus the need for costly second-line treatment); and (iii) a high-accuracy test has a greater impact than a moderate-accuracy test as a result of its higher sensitivity (Figs 2–4).

We have conservatively designated the confirmatory tests as ‘smear-like’ and ‘Xpert-like’, in recognition that performance data drawn from meta-analyses of clinical trials (Table 1) do not necessarily reflect the numbers of appropriate or erroneous TB treatments that would arise in real-world ACF implementation. In light of this uncertainty, the key conclusion of our analysis is not that one test should be preferred over another, but rather that specificity in field ACF conditions—for any diagnostic test—is a critical data gap to address for future ACF planning. In recent years the availability of molecular diagnostics has rapidly expanded for routine TB services in India [20–22]; our findings support the use of similar tests, with equal or higher specificity and the ability to detect rifampicin resistance, in ACF.

### Quantifying specificity

We note that quantifying ‘true’ specificity is a complex challenge, partly as the culture reference standard (against which specificity is judged) also has imperfect sensitivity, raising the question of how to interpret smear-positive, culture-negative results, particularly amongst those with strong clinical signs of TB. As initial steps in this direction, future ACF implementation research could aim, for example, to supplement microbiological reference standards with ‘composite’ reference standards that additionally incorporate clinical diagnosis and patient assessment on follow-up, including response to anti-TB treatment. Our results also have implications beyond the particular diagnostic tests being examined here. For example, a new generation of molecular diagnostic test, Xpert Ultra, has higher sensitivity than Xpert, but also lower specificity [6]. Its use in ACF would lead to similarly unacceptable numbers of false-positive TB treatments, as those illustrated in Fig 5A.

### Role of screening

Our sensitivity analysis shows that the performance of the screening stage can also play an influential role, although whether this widens or narrows the gap between the 2 diagnostic tests (at a given budget) depends on the relative magnitudes of (i) the reduction in false-positive TB treatments arising from a higher-specificity screening algorithm and (ii) the long-term second-line TB treatment costs that can be averted by a high-accuracy test (Fig C and Table A in S1 Text). In our current work, the overall effect is for our qualitative findings to remain unchanged. Strategies such as X-ray screening (not modelled in the current study) can show greater sensitivity and specificity than symptom screening alone [23], while allowing TB detection amongst those not reporting symptoms. Implementation of X-ray screening is more resource-intensive than symptom screening, but could be facilitated by the use of mobile radiography units [24,25], along with newly emerging technology for automated X-ray reads [26,27]. While the present analysis has focused on confirmatory tests, a more systematic exploration of these and other screening strategies will be an important area for future work.

### The patient perspective

Focusing on programmatic costs, our analysis does not address the important issue of patient costs associated with TB [28–30]. In the context of routine TB services, the costs of care-seeking and TB treatment can have a substantial impact on productivity and household income, and are an important cause of catastrophic health expenditure [29]. Previous work in India has shown that ACF can bring about substantial reductions in these patient costs [18], essentially by bringing TB services to those in need in a timely way. By neglecting patient costs, our analysis therefore does not capture the societal cost savings that would result from higher-sensitivity testing strategies. Our analysis also does not address the societal costs of false-positive TB diagnosis, including the potentially life-changing impact of stigma [13] and the potential side effects of TB treatment. Our analysis thus does not capture the societal cost savings that would result from higher-specificity ACF approaches. Overall, therefore, we expect that inclusion of societal costs would act to widen the gap between the strategies shown in Fig 3. The extension of our analysis to incorporate these important costs to the patient is an important area for future work.

### Key considerations in intervention costs

Our study has focused on the performance of confirmatory tests in ACF but has not addressed the implementation of these tests. For example, the overall cost of an Xpert test will depend on whether Xpert units are readily accessible for use in ACF, or whether they are only available in central laboratories, requiring the additional expense of sample transport and relaying test results to the patient. Previous work has addressed these considerations in the context of routine TB services [31]. Although such considerations are outside the scope of our current analysis, we note that access to Xpert facilities is likely to be a more pressing concern in peripheral health facilities in rural India than in urban slums. India’s current National Strategic Plan includes measures to improve the capacity for Xpert testing nationwide [32]. Moreover, recent developments offer prospects for reduced reliance on laboratory infrastructure, for example with the development of new, more mobile molecular diagnostics such as Xpert Omni [33] and Truenat [34], as well as the deployment of Xpert MTB/RIF through mobile diagnostic vans [35]. All of these developments would tend to reduce the per-test cost of high-accuracy diagnostic tools, by allowing closer proximity of testing to the ACF intervention. For any study aiming systematically to estimate the full cost of ACF, these and other implementation factors will be important to take into account (e.g., as demonstrated in [17,31]). Nonetheless, our findings illustrate an important consideration in any such study: In addition to these important cost components, the specificity of the confirmatory test, along with that of the whole ACF cascade, is likely to be a key driver of the cost/impact ratio of any ACF intervention.

Our analysis has ignored intervention costs directly associated with the rollout of the intervention, such as human resource costs and costs associated with the purchase of equipment. We also take a simple approach of assuming unit costs that are unaffected by the scale of the intervention, thus ignoring the potential for economies of scale, especially at high levels of population coverage. If human resource costs are similar under the 2 strategies, they would have little effect on the relative costs of these strategies. Overall, however, we emphasise that our estimates should not be interpreted as estimates of the actual cost of ACF. An important area for future work would be to incorporate these important elements [17,36], for more comprehensive estimates of the full cost of ACF.

### Model limitations

For simplicity, we ignored pre-treatment loss to follow-up. Although commonly observed in practical implementations of ACF [37], we do not expect this simplification to alter our essential findings, as long as it affects the 2 testing strategies roughly equally. Again, for simplicity, we assume that all false-positive TB diagnoses have the same rates of treatment completion as true-positive diagnoses; if their completion rates are substantially lower, this would tend to reduce the treatment cost associated with false-positive TB diagnosis. Finally, we note that there is considerable uncertainty about the potential transmission impact of ACF interventions, owing partly to a lack of direct evidence, particularly in South-Asian settings [4]. In the present analysis, our impact projections are based on a series of assumptions, including perfect implementation of symptom screening and diagnosis, high-quality engagement with and participation by the community, effective linkage to treatment, immediate rapid bacteriological suppression upon treatment (thus interrupting transmission), and assumptions about the degree to which ACF is able to identify TB cases while they still have substantial potential for passing on infection [4]. Lessons from current and future ACF initiatives will be invaluable in identifying which of these assumptions needs most attention.

## Conclusion

As ACF efforts are scaled up in India and other high-burden settings, implementation planning could benefit from a population perspective of potential costs and benefits. Such a perspective, complementing other approaches such as operational research, takes into account both the epidemiological impact and unintended consequences (such as false-positive diagnosis) of large-scale deployment. Addressing important questions about the optimum implementation of ACF could open the way for considerable impact on TB burden, in India and elsewhere.

## Supporting information

S1 CHEERS Checklist (DOC)

S1 Text Supporting information. Table A: Comparison of different screening algorithms and testing strategies. Table B: Proportion contribution of each cost component to the total incremental service cost. Fig A: Results of the model calibration. Fig B: Simulated impact and cost-effectiveness under alternative symptom screening strategies. Fig C: Breakdown of the ACF incremental service cost under the prolonged cough screening strategy. Fig D: Sensitivity analysis to different scenarios.
(DOCX)

## Abbreviations

ACF: active case finding
DR-TB: drug-resistant tuberculosis
DS-TB: drug-susceptible tuberculosis
NTS: non-TB symptomatic
TB: tuberculosis
