# Supplementary material for: Trade-offs between cost and accuracy in active case finding for tuberculosis: A dynamic modelling analysis
Source: PLoS Med. 2020 Dec 2;17(12):e1003456. doi: 10.1371/journal.pmed.1003456 (PMC7710036; doi:10.1371/journal.pmed.1003456)
Supplement: S1 Text — Table A: Comparison of different screening algorithms and testing strategies. Table B: Proportion contribution of each cost component to the total incremental service cost. Fig A: Results of the model calibration. Fig B: Simulated impact and cost-effectiveness under alternative symptom screening strategies. Fig C: Breakdown of the ACF incremental service cost under the prolonged cough screening strategy. Fig D: Sensitivity analysis to different scenarios. (DOCX) [file pmed.1003456.s002.docx]

**Trade-offs between cost and accuracy in active case-finding for tuberculosis: a dynamic modelling analysis**

**Supplementary Information**

Table of Contents

[1. Model technical details 2](#_Toc54866650)

[2. Model calibration 5](#_Toc54866651)

[3. Sensitivity analysis of the screening strategy 6](#_Toc54866652)

[4. Additional sensitivity analyses 9](#_Toc54866653)

[5. Additional tables 11](#_Toc54866654)

[References 12](#_Toc54866655)

## **Model technical details**

The overall model structure is illustrated in **Fig 1**, main text. To specify the model, we define the following subscripts:

$$q=\left\{ \begin{aligned} 0, DS TB \\ 1, DR TB \end{aligned} \right.$$

$$r=\left\{ \begin{aligned} 0, smear negative TB \\ 1, smear positive TB \end{aligned} \right.$$

$$s=\left\{ \begin{aligned} 0, asymptomatic TB \\ 1, symptomatic TB \end{aligned} \right.$$

$$u=\left\{ \begin{aligned} 0, public sector \\ 1, private sector \end{aligned} \right.$$

The full system of governing equations is as follows, with parameters as defined in **Table 1** in the main text:

Susceptible stage

$$\frac{dU}{dt}=b-\lambda_{q}U-\mu U,$$

Latent stages, slow progressors, $L_{q}$

$$\frac{dL_{q}}{dt}=\left( 1-p_{Fast} \right)\lambda_{q}U-aL_{q}-\mu L_{q},$$

Diseased and infectious stages, $I_{qrs}$

$$\frac{dI_{q00}}{dt}=\left( 1-\omega_{+} \right)p_{Fast}\lambda_{q}U+a\left( 1-\omega_{+} \right)L_{q}+\left( 1-\omega_{+} \right)\left( r_{1}R_{q}^{(lo)}+r_{2}R_{q}^{(hi)}+{r_{3}R}_{q} \right)+d_{Sx}A_{q00}^{(S)}+\left( 1-s_{0} \right)d_{Dx}A_{q00}^{(C)}-\left( m_{1}+e_{1}+\gamma+\mu+\mu_{TB}+k \right)I_{q00},$$

$$\frac{dI_{q10}}{dt}=\left( p_{Fast}\omega_{+} \right)\lambda_{q}U+a\omega_{+}L_{q}+\omega_{+}\left( r_{1}R_{q}^{(lo)}+r_{2}R_{q}^{(hi)}+{r_{3}R}_{q} \right)+m_{1}I_{q00}+d_{Sx}A_{q10}^{(S)}+\left( 1-s_{1} \right)d_{Dx}A_{q10}^{(C)}-\left( e_{0}+\gamma+\mu+\mu_{TB}+k \right)I_{q10},$$

$$\frac{dI_{q01}}{dt}=e_{1}I_{q00}+\left( 1-s_{0} \right)d_{Dx}A_{q01}^{(C)}-\left( m_{0}+\gamma+\mu+\mu_{TB}+r_{CS}+k \right)I_{q01},$$

$$\frac{dI_{q11}}{dt}=e_{0}I_{q10}+m_{0}I_{q01}+\left( 1-s_{1} \right)d_{Dx}A_{q11}^{(C)}-\left( \gamma+\mu+\mu_{TB}+r_{CS}+k \right)I_{q11},$$

Diseased and infectious stages, $E_{qr}$, for those who were mis-diagnosed or mis-treated by passive health-care system; assumed to be symptomatic

$$\frac{dE_{00}}{dt}=\left( 1-p_{u}^{\left( Dx \right)}p_{u}^{\left( Tx \right)} \right)d_{Dx}D_{00u}+[\left( 1-c_{u}^{1} \right)+r_{def1}]d_{TxFL}T_{00u}^{(FL)}+\left( 1-s_{0} \right)d_{Dx}A_{001}^{(C)}-\left( m_{0}+\gamma+\mu+\mu_{TB}+r_{cs}^{\left( 2 \right)}+k \right)E_{00},$$

$$\frac{dE_{10}}{dt}=\left( 1-p_{u}^{\left( Dx \right)}p_{u}^{\left( Tx \right)} \right)d_{Dx}D_{10u}+(1-p_{SL})\left( 1-c_{u}^{1} \right)d_{TxFL}T_{10u}^{(FL)}+\left( 1-s_{0} \right)d_{Dx}A_{101}^{(C)}-\left( m_{0}+\gamma+\mu+\mu_{TB}+r_{cs}^{\left( 2 \right)}+k \right)E_{10},$$

$$\frac{dE_{01}}{dt}=\left( 1-p_{u}^{\left( Dx \right)}p_{u}^{\left( Tx \right)} \right)d_{Dx}D_{01u}+\left[ \left( 1-c_{u}^{1} \right)+r_{def1} \right]d_{TxFL}T_{01u}^{\left( \mathrm{FL} \right)}+\left( 1-s_{1} \right)d_{Dx}A_{011}^{\left( C \right)}+m_{0}E_{00}-\left( \gamma+\mu+\mu_{TB}+r_{cs}^{\left( 2 \right)}+k \right)E_{01},$$

$$\frac{dE_{11}}{dt}=\left( 1-p_{u}^{\left( Dx \right)}p_{u}^{\left( Tx \right)} \right)d_{Dx}D_{11u}+\left( 1-p_{SL} \right)\left( 1-c_{u}^{1} \right)d_{TxFL}T_{11u}^{\left( \mathrm{FL} \right)}+\left( 1-s_{1} \right)d_{Dx}A_{111}^{\left( C \right)}+m_{0}E_{10}-\left( \gamma+\mu+\mu_{TB}+r_{cs}^{\left( 2 \right)}+k \right)E_{11},$$

Active case-finding stages, screening $A_{qrs}^{(S)}$

$$\frac{dA_{q00}^{(S)}}{dt}=\left( \frac{q}{q_{0}} \right)kI_{q00}-\left( e_{1}+m_{1}+d_{Sx}+\gamma+\mu+\mu_{TB} \right)A_{q00}^{(S)},$$

$$\frac{dA_{q10}^{(S)}}{dt}=\left( \frac{q}{q_{0}} \right)kI_{q10}+m_{1}A_{q00}^{(S)}-\left( e_{0}+d_{Sx}+\gamma+\mu+\mu_{TB} \right)A_{q10}^{(S)},$$

$$\frac{dA_{q01}^{(S)}}{dt}=\left( \frac{q}{q_{0}} \right)k\left( I_{q01}+E_{q0} \right)+e_{1}A_{q00}^{(S)}-\left( m_{0}+d_{Sx}+\gamma+\mu+\mu_{TB} \right)A_{q01}^{(S)},$$

$$\frac{dA_{q11}^{(S)}}{dt}=\left( \frac{q}{q_{0}} \right)k\left( I_{q11}+E_{q1} \right)+e_{0}A_{q10}^{(S)}+m_{0}A_{q01}^{(S)}-\left( d_{Sx}+\gamma+\mu+\mu_{TB} \right)A_{q11}^{(S)},$$

Here, *k* represents the intervention coverage, i.e. the proportion of the slum population being screened every year, while $q$ is the sensitivity of the screening algorithm, relative to the sensitivity $q_{0}$ of an algorithm to identify any symptoms suggestive of TB. The reason for this designation is that, when we calibrate the model to the proportion of TB being symptomatic, we interpret this as the proportion having *any* TB symptoms, that is using the broadest possible symptom definition (see **Table 2**, main text). These individuals would, by definition, all screen positive under an algorithm identifying ‘any TB symptoms’ (i.e. $q/q_{0}$ = 1). Moreover, only a subset would screen positive under a more restrictive symptom definition, proportional to the sensitivity $q$of that symptom definition.

Active case-finding stages, confirmatory diagnosis $A_{qrs}^{(C)}$; only individuals screening positive under symptom screening $(s=1)$ continue to the confirmatory diagnostic stage, while asymptomatic individuals return to their respective $I_{qrs}$ compartments. Therefore compartments $A_{q00}^{(C)}$ and $A_{q10}^{(C)}$ remain empty, and:

$$\frac{dA_{q01}^{(C)}}{dt}=d_{Sx}A_{q01}^{(S)}-\left( m_{0}+d_{Dx}+\gamma+\mu+\mu_{TB} \right)A_{q01}^{(C)},$$

$$\frac{dA_{q11}^{(C)}}{dt}=d_{Sx}A_{q11}^{(S)}+m_{0}A_{q01}^{(C)}-\left( d_{Dx}+\gamma+\mu+\mu_{TB} \right)A_{q11}^{(C)},$$

Passive health-care diagnosis and treatment (first- and second-line) stages, $D_{qru}$ , $T_{qru}^{\mathrm{FL}}$ and $T_{qru}^{\mathrm{SL}}$

$$\frac{dD_{qru}}{dt}=r_{CS}p_{u}I_{qr1}+r_{CS}^{\left( 2 \right)}{p_{u}E}_{qr}-\left( d_{Dx}+\gamma+\mu+\mu_{TB} \right)D_{qpr},$$

$$\frac{dT_{0ru}^{(FL)}}{dt}=d_{Dx}s_{r}A_{0rs}^{(C)}+d_{Dx}p_{u}^{\left( Dx \right)}p_{u}^{\left( Tx \right)}D_{0ru}-\left( d_{TxFL}+r_{MDR}+\gamma+\mu\right)T_{0ru}^{(FL)},$$

$$\frac{dT_{1ru}^{(FL)}}{dt}=(1-p_{DSTA})d_{Dx}s_{r}A_{1rs}^{(C)}+p_{u}^{\left( Dx \right)}p_{u}^{\left( Tx \right)}\left( 1-p_{DST}^{u} \right)D_{1ru}+r_{MDR}T_{0ru}^{(FL)}-\left( d_{TxFL}+\gamma+\mu\right)T_{1ru}^{(FL)},$$

$$\frac{dT_{1ru}^{(SL)}}{dt}=p_{DSTA}d_{Dx}s_{r}A_{1rs}^{(C)}+d_{Dx}p_{u}^{\left( Dx \right)}p_{u}^{\left( Tx2 \right)}p_{DST}^{u}D_{1ru}+(1-c_{0}^{1})d_{TxFL}p_{SL}T_{0ru}^{(FL)}-\left( \gamma+\mu+d_{TxSL} \right)T_{1ru}^{(SL)},$$

Relapse stages, high/low depending on treatment default/success and long-term, $R_{q}^{(lo)}$/$R_{q}^{(hi)}$ and $R_{q}$

$$\frac{dR_{0}^{(hi)}}{dt}=\gamma\sum(I_{0rs}+E_{0r}+D_{0ru}+A_{0rs}^{\left( S \right)}+A_{0rs}^{\left( C \right)}+T_{0ru}^{\left( FL \right)})+r_{def1}^{u}T_{0ru}^{(FL)}-\left( \mu+r_{2}+0.5 \right)R_{0}^{(hi)},$$

$$\frac{dR_{1}^{(hi)}}{dt}=\gamma\sum(I_{1rs}+E_{1r}+D_{1ru}+A_{1rs}^{\left( S \right)}+A_{1rs}^{\left( C \right)}+T_{1ru}^{\left( FL \right)}+T_{1ru}^{\left( SL \right)})+r_{def2_{u}}^{u}T_{1ru}^{(SL)}-\left( \mu+r_{2}+0.5 \right)R_{1}^{(hi)},$$

$$\frac{dR_{0}^{(lo)}}{dt}=d_{TxFL}c_{u}^{1}T_{0ru}^{(FL)}-\left( \mu+r_{1}+0.5 \right)R_{0}^{(lo)},$$

$$\frac{dR_{1}^{(lo)}}{dt}=d_{TxSL}c_{2}T_{1ru}^{(SL)}-\left( \mu+r_{1}+0.5 \right)R_{1}^{(lo)},$$

$$\frac{dR_{q}}{dt}=0.5(R_{q}^{(hi)}+R_{q}^{(lo)})-(\mu+r_{3})R_{q},$$

For $\lambda_{q}=\beta_{q}[I_{q1s}+E_{q1}+D_{q1u}+A_{q1s}^{(S)}+A_{q1s}^{(C)}+\varepsilon\left( I_{q0s}+E_{q0}+D_{q0u}+A_{q0s}^{(S)}+A_{q0s}^{(C)} \right)]$, where $\varepsilon$ represents the relative reduction of infectious of smear-negative TB compared to smear-positive TB (see **Table 1**, main text); $k$ represents the intervention coverage, i.e. the proportion of the slum population being screened every year; $p_{u}$ represents the proportion seeking care from the public $(u=0)$ or private $(u=1)$ sector; $p_{DSTA}$ represents the proportion of DR-TB recognised by the diagnostic test used in ACF intervention.

**Non-TB symptomatic population**

For the purpose of counting false-positive diagnoses, we defined a non-TB symptomatic (NTS) population, as individuals who do not have TB, but who would screen positive under a given screening algorithm. Assuming this population to be independent of the TB population described above, we modelled its dynamics under an active case-finding intervention, assuming the same rate of screening as the TB population. Governing equations for the NTS population are as follows:

Symptomatics not undergoing screening, testing or current TB treatment (S):

$$\frac{dS}{dt}=d_{TxFL}\sigma A^{(C)}+r_{1}T-kS,$$

Symptomatics undergoing symptom screening ($A^{(S)}$):

$$\frac{dA^{(S)}}{dt}=kS-d_{Dx}A^{(S)},$$

Symptomatics screening positive and undergoing confirmatory testing ($A^{\left( C \right)}$):

$$\frac{dA^{(C)}}{dt}=d_{Dx}A^{(S)}-d_{TxFL}A^{(C)},$$

Symptomatics testing (false) positive on confirmatory test and undergoing first-line TB treatment ($T$):

$\frac{dT}{dt}=d_{TxFL}\left( 1-\sigma\right)A^{(C)}-r_{1}T$.

where $\sigma_{C}$ is the specificity of the confirmatory test, which determines the number of false-positive diagnoses.

Because every negative term in these equations is balanced by a corresponding positive term, the total size of the NTS population remain constant throughout the simulation. Indeed, the size of this population is determined by the specificity of the screening algorithm, $\sigma_{S}$: it is fixed at a value of $N(1-\sigma_{S})(1-p)$, where *N* is the overall size of the population in the TB governing equations; and $p$ is the TB prevalence.

## **Model calibration**

Under a set of given parameters, we first simulated the model to endemic equilibrium, in the absence of the public sector and DR-TB, assuming these conditions to hold until 1970. After 1970, we simulated population growth at a rate of 2.4% per year (through an increase in the birth rate), as well as the introduction of DR-TB, as a result of the introduction of rifampicin-based treatment. From 1990 to 2007 we simulated the scale up of the Revised National Tuberculosis Control Program (RNTCP) via the adoption of DOTS, by assuming a linear increase in the parameter $p_{0}$ (the proportion of symptomatics visiting the public sector) from zero to $1-$ the value given in **Table 1** in main text ($p_{1}$, the proportion visiting the private sector instead). The model was finally simulated to 2018 and we compared the model outputs for prevalence, annual risk of TB infection and other indicators against data reflecting conditions consistent with urban slums in India (see **Table 2** in main text for these calibration targets). **Fig A** shows the resulting model calibration.

Using Latin hypercube sampling, we propagated uncertainty in a simple way by sampling model parameters from their respective ranges (10,000 samples) and simulating the model to 2018 as described above. We assessed model projections for prevalence in 2012 and for ARTI in 2006, consistent with the dates of the relevant data (**Table 2** main text). Parameter sets that yielded 2018 projections within the calibration target ranges were accepted, and others were rejected. The set of accepted parameters was then used to calculate the 95% credible intervals.

**
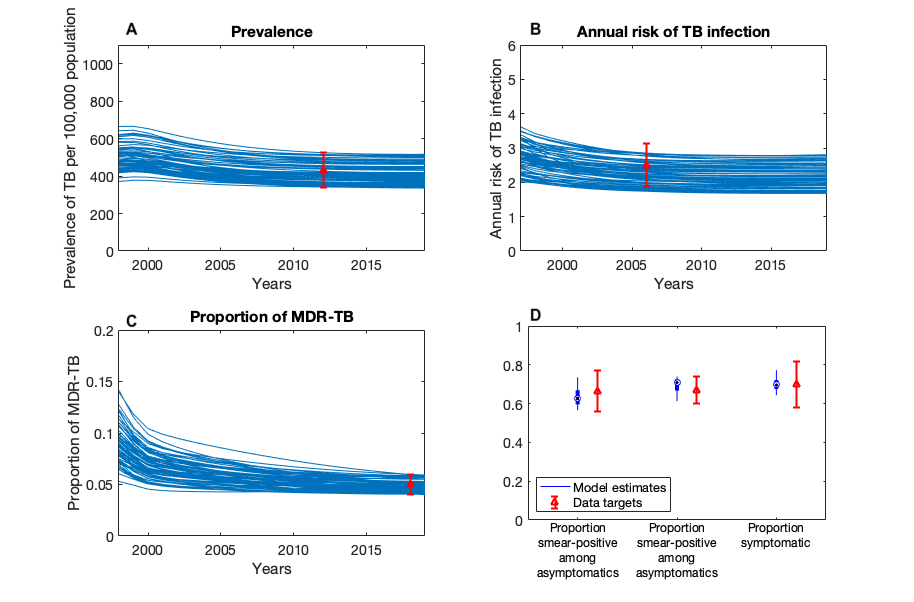
**

**Fig A. Results of the model calibration for the six calibration targets outlined in Table 2, main text.** The red lines in panels (A)-(C) represent data (calibration targets), drawn from prevalence survey data (1). In panel (D) the calibration targets are shown by coloured triangles.

## **Sensitivity analysis of the screening strategy**

Analysis in the main text assumes symptom screening based on ‘any TB symptom’ as an eligibility criterion, having sensitivity and specificity of 70% and 60% respectively. As a sensitivity analysis, we also conducted model simulations when assuming instead the use of ‘prolonged cough’ as a screening algorithm, a more restrictive condition having sensitivity and specificity of 25% and 96% respectively.

Thus, in the above governing equations, we took $q/q_{0}=0.36$to represent the diminished sensitivity of this algorithm. To represent the improved specificity, we assumed an NTS population of size 4% of the overall population used for modelling TB dynamics (see p.4 for a description of how the NTS population determines the specificity of the screening algorithm).

**Fig B** shows results under this alternative symptom screening strategy (solid lines), also showing main text results (dashed lines) for comparison. The left-hand panel shows impact as a function of ACF coverage, or the proportion of the population screened per year. As would be expected, the reduced sensitivity at the screening stage has the effect of reducing the overall impact of ACF, whether by high-accuracy or moderate-accuracy strategies (left-hand panel). However, the right-hand panel shows the impact that could be achieved under a given budget, or fixed level of incremental spending between 2020 and 2035. Here, the impact when using a prolonged cough screening approach is greater than the ‘any symptom’ approach presented in the main text. **Table A** illustrates why: the ‘prolonged cough’ scenario requires a substantially higher coverage of annual screening, in order to meet a budget of USD 20million. Thus the dashed and solid lines in **Fig** **B**, right-hand panel, represent two very different levels of coverage, under a given budget (for example, as represented by the dashed vertical line).

**Fig C** illustrates implications for incremental spending, illustrating the strong changes that are induced, when adopting prolonged cough as a screening algorithm, while keeping the budget fixed. On the one hand the false-positive TB treatments for both testing strategies are substantially reduced, while on the other, the impact of the high-accuracy test on second-line costs is intensified. Overall, therefore, the effect is to broaden the separation between the high- and moderate-accuracy tests, in their relative impact at this reference budget of USD 20 million. For prolonged cough as a screening approach, this relative impact is 1.28 (95% simulation intervals 0.91 – 2.02), compared with 1.14 (0.75 – 1.99) reported in the main text. However, we caution that this broadened separation, resulting from a more-specific screening algorithm, is not necessarily a general result: it is likely to be contingent on the various cost components in the model, as well as on additional intervention costs that we have not considered here, that would be incurred in expanding the coverage of ACF screening by the amounts shown in **Table A**.

| Screening strategy | Testing strategy | Annual screening coverage | Cases averted  (per 100,000) | Relative impact |
| --- | --- | --- | --- | --- |
|  |  |  |  |  |
| **Any symptom** | **Moderate accuracy** | 6% | 17 | 1.27 |
|  | **High accuracy** | 7% | 22 |  |
| **Prolonged cough** | **Moderate accuracy** | 52% | 41 | 1.57 |
|  | **High accuracy** | 77% | 65 |  |


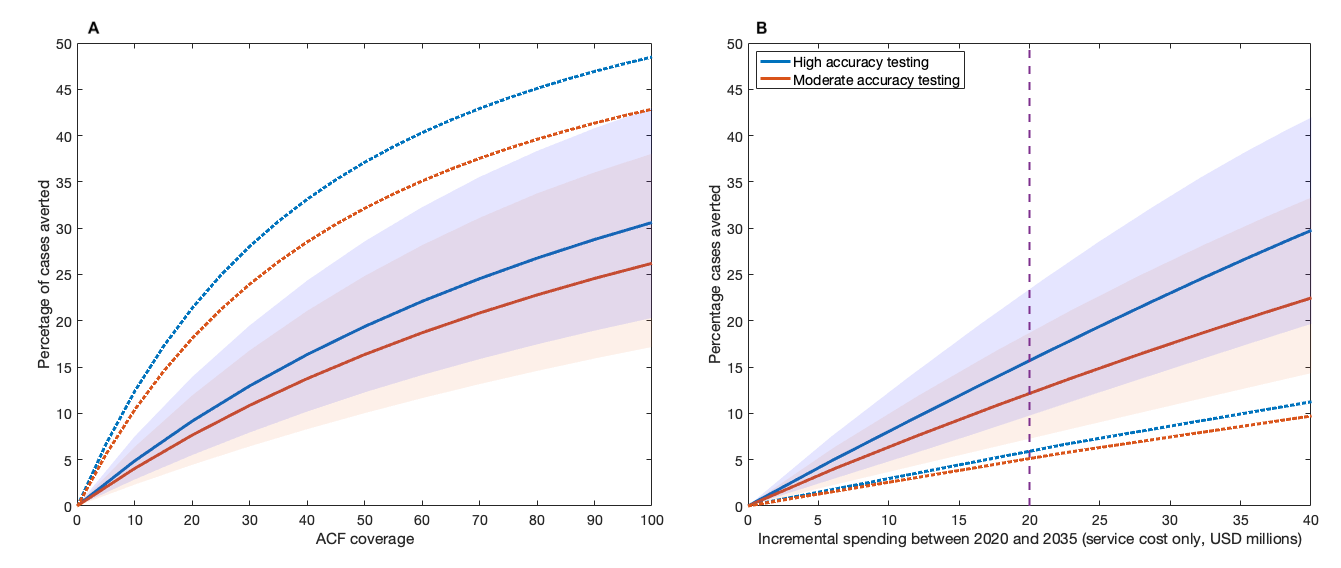


**Fig B. Simulated impact and cost-effectiveness under alternative symptom screening strategies.** Solid lines show results when using ‘prolonged cough’ as a screening criterion, while (for comparison) dashed lines show results presented in the main text, when using ‘any TB symptoms’ as a criterion. Shaded intervals show uncertainty intervals for the prolonged cough scenario. For clarity, we omit uncertainty intervals for the ‘any symptom’ scenario.

**Table A. Comparison of different screening algorithms and testing strategies, at a fixed budget of USD 20 million between 2020 and 2035.** The ‘prolonged cough’ screening strategy has a lower sensitivity for TB than ‘any symptom’, but this also means that it requires a substantially higher screening coverage in order to meet a budget of USD 20 million (‘annual screening coverage’ column), thereby leading to a greater impact overall (‘cases averted’ column).


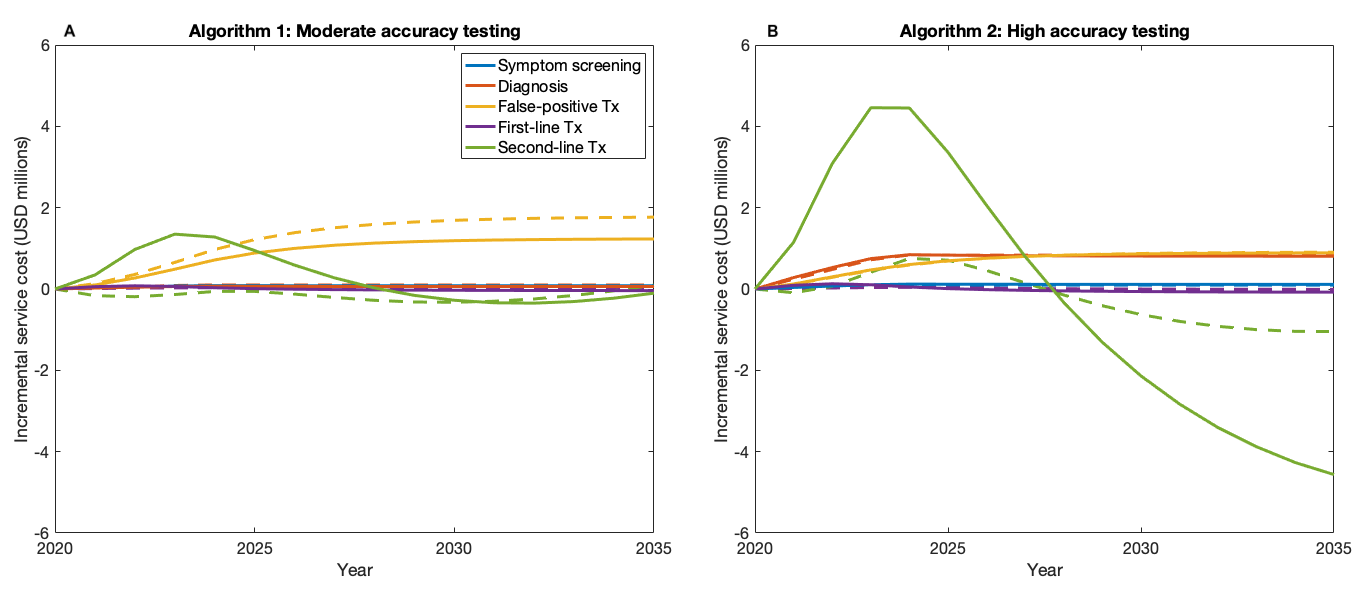


**Fig C. Breakdown of the ACF incremental service cost under the prolonged cough screening strategy,** at fixed total incremental cost of USD 20 million. The dashed lines show, for comparison, the cost components under the main text screening strategy, any symptoms.

## **Additional sensitivity analyses**

*Alternative scenarios*

Once again adopting the focal model output used in **Fig 4** in the main text (i.e. the relative impact of high- vs moderate-accuracy strategies under an incremental spend of USD 20 million), we calculated this output under the following alternative scenarios:

- Where the sensitivity of smear microscopy is 25% and 75% respectively, for smear-negative and smear-positive TB (rather than 0% and 100% as assumed in the main text)
- Where the symptom screening involves ‘prolonged cough’ rather than ‘any TB symptom’ (as shown in **Fig B** and accompanying description).
- Where second-line treatment success is increased from 50% to 75%, as a result of adoption of new, shorter regimens for drug-resistant TB
- Where the cost of second-line treatment is reduced by 50%, again as a result of future adoption of new regimens
- When the burden of DR-TB is 15% of incident TB (as opposed to 5% in the main text), to address cities such as Mumbai having a concentrated burden of DR-TB.

Results are shown in **Fig D**. The result under ‘prolonged cough as symptom screening’ corresponds to **Fig B**, right-hand panel, illustrating the important role of the specificity of symptom screening. Indeed, additional analysis (not shown in **Fig D**) shows that a screening specificity of 97.5% is sufficient for there to be no significant difference in cost-effectiveness between high-accuracy and moderate-accuracy testing strategies.

Notable cases include the scenario where the sensitivity of smear is assumed to be 25% and 75% respectively, of smear-negative and smear-positive TB; that is, an increase in sensitivity for smear-negative TB, and a decrease for smear-positive TB, relative to the scenario presented in the main text. Because smear-positive TB is assumed to be more infectious than smear-negative, the net effect is for a smear-based ACF that is less sensitive than that presented in the main text, thus widening the gap in impact between the two testing strategies.

Another notable case is that of an increased burden of drug-resistant TB; the management of DR-TB accounts for a disproportionate share of programmatic costs. ACF can increase this spending by increasing the number of drug-resistant cases being detected and put on second-line treatment, but in the long term, may lead to overall cost savings arising from a diminished burden of DR-TB. These results illustrate that these cost savings are likely to be stronger in settings with a higher DR-TB burden.


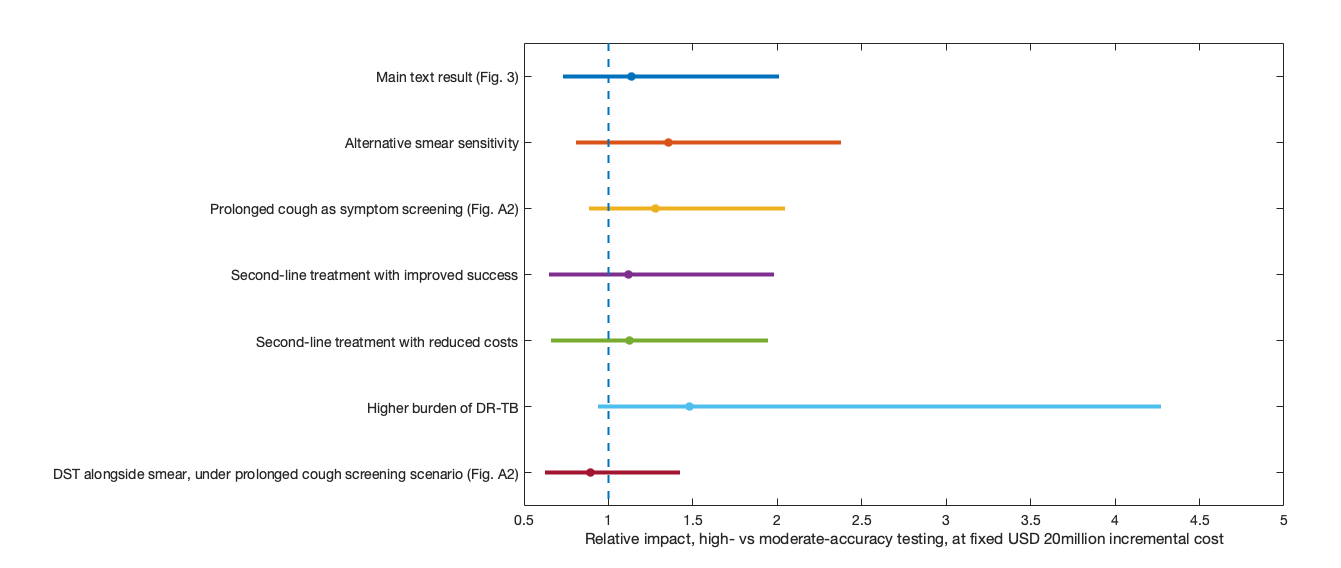


**Fig D. Sensitivity analysis to different scenarios.** As in **Fig 4** in the main text, as a focal model output, we select the relative impact of high- vs moderate-accuracy testing, at an incremental spend of USD 20 million. The region to the right of the dotted line represents high-accuracy testing being more cost-efficient than moderate-accuracy, and vice versa. Dots show central estimates, while lines show 95% credible intervals. Parameters for each sensitivity scenario are listed in the supplementary text above.

## **Additional tables**

| Cost component | Proportion contribution to total incremental  service cost, 2020-2035 (95% credible interval) | |
| --- | --- | --- |
|  | Smear | Xpert |
| Symptom screening | 4% (3%-11%) | 5% (3%-8%) |
| Diagnosis | 5% (3%-12%) | 42% (22%-72%) |
| False-positive treatment (first-line) | 76% (42%-90%) | 37% (6%-61%) |
| True-positive first-and second-line treatment | 14% (1%-47%) | 14% (1%-44%) |

**Table B. Proportion contribution of each cost component to the total incremental service cost,** at 50% population coverage, under the “any symptom” screening scenario presented in the main text.

## **References**

1. Dhanaraj B, Papanna MK, Adinarayanan S, Vedachalam C, Sundaram V, Shanmugam S, et al. Prevalence and risk factors for adult pulmonary tuberculosis in a metropolitan city of south India. Tyagi AK, editor. PLoS One [Internet]. 2015 Apr 23 [cited 2016 Nov 4];10(4):e0124260. Available from: http://dx.plos.org/10.1371/journal.pone.0124260
